# An Old Subject Revived

**Published:** 1896-08

**Authors:** 


### Editorial.

### AN OLD SUBJECT REVIVED.

It would seem a useless expenditure of mental strength to endeavor to counteract all the open attacks made on dental colleges and the National Association of Dental Faculties, and we recur to this subject with reluctance, but the short article in the present number on this subject seems to require an answer, not so much because of any new or a clearer statement of the question, so often discussed, as from the fact that it contains some oft-repeated views which must be regarded by experienced teachers as radically wrong, and yet these are held by a very large number of the most progressive minds in the dental profession.

In an article in a previous number, it was asserted that the tendency of dental teaching was towards a medical degree as a prerequisite to the dental. It is still believed that absorption in the medical will be, right or wrong, the ultimate end of the dental profession.

It is unnecessary to recall the history of dental work the world over. It has been an independent movement, and has gradually developed from crude beginnings to a position in which there would seem to be a necessity for a consideration of the question in its intricate relations to medical science. The change, if change be made, will not come through the writing of essays or caustic remarks on the present methods of colleges. It would be a useless waste of time to attempt to argue that dental colleges are perfect; indeed, the entire scheme of education in this country is very far from the state of almost perfection reached in some foreign countries. It is freely acknowledged that dental education is in process of evolution, and, therefore, must of necessity be in a degree imperfect, for the word itself means a process of unfolding, an effort to reach a higher standard.

It is conceded that "a complete medical knowledge for the dentist creates a power of conception," or rather would were it possible to give any graduate, medical or dental, a complete knowledge of medicine in all its branches. This must be recognized as an impossibility. Medicine to-day is made up of a series of important specialties. The general practitioner transfers his patient, when the case is beyond his skill, to the surgeon, ophthalmologist, otologist, gynæcologist, laryngologist, etc., nor would he be justified in treating such cases.

The argument of the essayist, and such as hold with him, is, that "the dental student should only be taken from the ranks of the medical profession." What constitutes the basis of medical instruction? First, a fairly accurate knowledge of human anatomy, physiology, pathology, chemistry, histology, materia medica, pharmacy, obstetrics, and surgery. These are regarded, very properly, as foundation studies, others elective. If, then, this be true, can this knowledge be acquired outside the walls of the medical college proper? Is there anything in the atmosphere there that gives the student an indefinable something not to be acquired or experienced in other schools teaching the same branches? Is there anything in the degree of Doctor of Medicine which will have a tendency to make a man more profoundly learned in the science, if it be a science, of treating disease? The answer, it seems to the writer, must be in the negative. It is recognized that association in any line of work is of marked advantage to the individual and induces a professional enthusiasm not obtainable by the study elsewhere, but beyond this and the supposed dignity which accrues to a young man who can write M.D. after his name, there would seem to be little or nothing gained.

If the position be the true one in regard to the foundation studies, then the query naturally arises, Can these studies be made in dental colleges equally as well as in the medical? No one familiar with the best dental teaching of to-day would attempt to assert to the contrary, except in one instance, a subject not important to the dentist. It is probably true that in many of the dental colleges of the country these foundation subjects are indifferently taught and some are omitted altogether. The study of anatomy is not one to be confined to the head, but comprises the entire organism, and no reason exists why it should not be thoroughly taught in all dental schools. The kind of partial culture alluded to has long since been relegated to the superficial curriculum of the past. The effort at the present time, and to a very large degree successful, is to give a thorough training in all these foundation branches. This will, in the nature of things, require years to perfect, but the work of the best dental colleges, even as at present arranged, is so far removed from that of twenty-five years ago that comparison cannot be made.

"Our dental colleges are pursuing an entirely wrong course in the method of their teaching." Presuming that our critic is entirely familiar with the best dental colleges of the country, it would seem that he is in duty bound to explain wherein the course adopted by these institutions is wrong. The remedy is given in a line. "There should be but one profession, and that the medical."

The force of this is not so apparent as the essayist seems to imagine. It is not clear that the medical profession has any reserved right to absorb all the branches of human activity that impinge in a near or remote degree upon its supposed domain. On the contrary, it is surmised that independent work outside of the ruts of tradition, untrammelled by precedents of a thousand years, may produce a new and a higher form of energy outside of and not particularly obligated to the mother calling. It is a law of development that the offspring will find strength and more perfect growth in independent fields and will be equally weakened in proportion as it receives nutrition alone from the mother source. In illustration of the working of this law we may refer with confidence to the energy, freedom in application of new processes, enlarged growth of dentistry, untrammelled as it has been in a century of development, and compare this activity with that of the specialties of medicine that have been developed, as such, alongside of the parent stem.

Without continuing this line of argument further, it must have been clearly evident to practical observers that the dentistry of the present covers such a wide field in mechanics alone that any attempt to make the study of medicine as a prior requisite to the study of dentistry must result in failure. The work of the dentist is necessarily, in its foundation, a manual cultivation. The development of dexterity in handling tools must be acquired somewhere and somehow. This cannot be accomplished in a medical college. Start a young man of twenty in a medical college, fresh from his literary training in the schools, and oblige him to spend four years in the study of the fixed branches of a medical education, what will be the result? He may graduate at the end of that term at twenty-four with not the slightest practical knowledge of the work he is expected to follow,—that of the practice of dentistry. He then takes up the latter work for another course of three years, or, perhaps, with his credits, two years. This, it is conceded, is none too long, but how many men will make good practical dentists after twenty-four years? A very few naturally gifted may, perhaps, accomplish manual dexterity, but the majority would prove sad failures. This is the experience of the writer with that class of men, and, in the nature of things, it can never be otherwise. Progress is not made by retrogression, and the average man will regard the effort to study dentistry after a full course in medicine in that light, and however he may desire to acquire it, dignity and lack of practical training steps in and rebels.

The true course, in the opinion of the writer, is to make dental colleges thorough in all the foundation branches of a medical education, and the higher schools are rapidly approaching this standard. The work in these institutions has become so severe that the question is beginning to be heard in the faculties, "How are we to teach dentistry with the students overloaded with strictly medical studies?" It is a serious problem and will have to be considered in the very near future. There seems no way out of the difficulty but by a lengthening of the course to four or even five years.

With the present curriculum of the best dental schools a young man will have no difficulty in taking the course in medicine as a post-graduate work, and those who do continue their studies further find a rich reward, not in the degree obtained, but in that additional mental training in the branches already taken.

Criticism is a valuable aid to progress when conducted in the right spirit and based on knowledge, but that which has its origin in prejudice and a superficial acquaintance with modern methods of work must not only fail, but be the cause of injury to the efforts already made to advance the standard of dental education. The dental colleges of the country are rapidly progressing to an advanced medical training, but this should be gained not by following traditions within medical walls but in the freer air of an independent curriculum.

